# Low dimensions electron localization in the beyond real space super cell approximation

**DOI:** 10.1038/s41598-019-44395-w

**Published:** 2019-06-04

**Authors:** Rostam Moradian, Sina Moradian

**Affiliations:** 1 Department of Physics, Faculty of Science Razi University, Kermanshah, Iran; 2 Nano science and nano technology research center, Razi University, Kermanshah, Iran; 3 Department of Electrical and Computer Engineering, University of Central Florida, Orlando, Florida USA

**Keywords:** Electronic properties and materials, Theoretical physics, Condensed-matter physics, Phase transitions and critical phenomena

## Abstract

Metal to insulator phase transition due to electron localization in disordered alloys (Anderson transition) and interacting electrons (Mott transition) systems is one of major problem in these fields. Multi site electron scattering is responsible for localization which can’t be seen by single site approximations such as coherent potential approximation (CPA) and dynamical mean field theory (DMFT). Here we develop a multi site technique to calculate multi site electron scattering for observation of phenomenons such as electron localization especially in low dimension systems. Our self-energy in first Brillouin zone (FBZ) is casual, in contrast to previous approximation fully crystal electron wave vector, **q**, dependent and continuous with respect to **q**. It recovers coherent potential approximation in the single site approximation and is exact when the number of sites in the super cell approaches to the total number of lattice sites. We illustrate that this approximation undertakes electrons localization for one and two dimensional alloy systems which isn’t observed by previous multi site approximations such as dynamical cluster approximation (DCA).

## Introduction

The treatment of disordered and interacting electron systems based on single electron motion in an effective medium is an important problem in many fields such as alloys, strongly correlated systems, magnetism and superconductivity in condensed matter physics. Coherent Potential Approximation (CPA) and Dynamical Mean Field Theory (DMFT) are single site approximations for calculating effective medium denoted by self-energy. In these approximations muti-site effects is neglected. Metzner and Vollhardt^1^ and Muler-Hartmann^2^ found that in the limit of infinite dimensions both single site approximations coherent potential approximation (CPA)^3^ for disordered system and dynamical mean field theory for interacting systems are exact^1,2^. This means self-energy for systems with high dimensions is k-independent. However, outside of systems with infinite dimensions especially in one and two dimensional systems self-energy is far from local which means it is k-dependent. To treat effective features of disorder systems, k-dependent relation of self-energy Σ(**k**; *E*) must be identified. In lower approximations such as Born approximation, T-matrix approximation, and Coherent Potential Approximation (CPA)^3^, which are single site approximations, self-energy is k-independent Σ(**k**; *E*) = Σ(*E*). In these approximations multi-site scattering is neglected which leads to overestimation band splitting and also losing short range effects. To add these effects cluster CPA with k-independent self-energy is used^4^. Dynamical Cluster Approximation (DCA)^5–7^ for systems with weak k-dependent self energies by considering periodic boundary condition for both interacting electron and disorder systems introduced. Also cellular dynamical mean field theory (CDMFT) approximation with open boundary condition^8^ was introduced. In DCA, first Brillouin zone (FBZ) is divided to *N*_*c*_ grain regions where self-energy inside of these grains is k-independent although they could be different. So their self-energy in the FBZ is not continues. The wave vectors at center of these grains called cluster wave vectors and are denoted by $\{{{\bf{K}}}_{1},\ldots ,{{\bf{K}}}_{{N}_{c}}\}$. For disordered systems they claimed these cluster wave vectors, {**K**_*n*_}, corresponds to a *N*_*c*_ real cluster sites^5^. Although both cluster approximations DCA and CDMFT are successful in importing multi site effects but these methods have two major weakness, first their grain self energies are discontinuous, second at low dimension self energy is strongly k-dependent. In real space effective medium super-cell approximation (EMSCA)^9,10^ are used to approximate self-energy of interacting disordered systems. Here in real space we first introduce super cell approximation by neglecting k-space self-energy contribution of all sites in different super cells. Then by keeping this contribution we go beyond super cell approximation. In our formalism, self energy is k-dependent and continuously varying in FBZ. Our self-energy is more close to real self-energy. Hence the average Green function calculated with our method used for calculation of physical quantities is more close to real average Green function.

The organization of the paper is as follows. In Sec. II the model Hamiltonian and super cell approximation equations are presented. Beyond super cell approximation equations derived and applied to a two dimensional alloy system in Sec. III. In this section we calculated and compared density of states for single site, super cell and beyond super cell approximations. Also electron localization in these approximations are discussed.

## Model Hamiltonian and Self-Energy in the Super Cell Approximation

The starting point is a tight-binding model for a disorder alloy system which is given by,

1 $$\begin{array}{c}H=-\,\sum _{ij\sigma \sigma }\,{t}_{ij}{c}_{i\sigma }^{\dagger }{c}_{j\sigma }+\,\sum _{i\sigma }({\varepsilon }_{i}-\mu ){c}_{i\sigma }^{\dagger }{c}_{i\sigma },\end{array}$$

where ${c}_{i\sigma }^{\dagger }$ (*c*_*iσ*_) is the creation (annihilation) operator of an electron with spin *σ* on lattice site *i* and ${\hat{n}}_{i\sigma }={c}_{i\sigma }^{\dagger }{c}_{i\sigma }$ is the number operator. ${t}_{ij}^{\sigma \sigma }$ are the hopping integrals between *i* and *j* lattice sites with spin *σ* respectively. *ε*_*i*_ is the random on-site energy and takes −*δ* with probability 1 − *c* for the host sites and *δ* with probability *c* for impurity sites and *μ* is the chemical potential.

**Figure 1:**
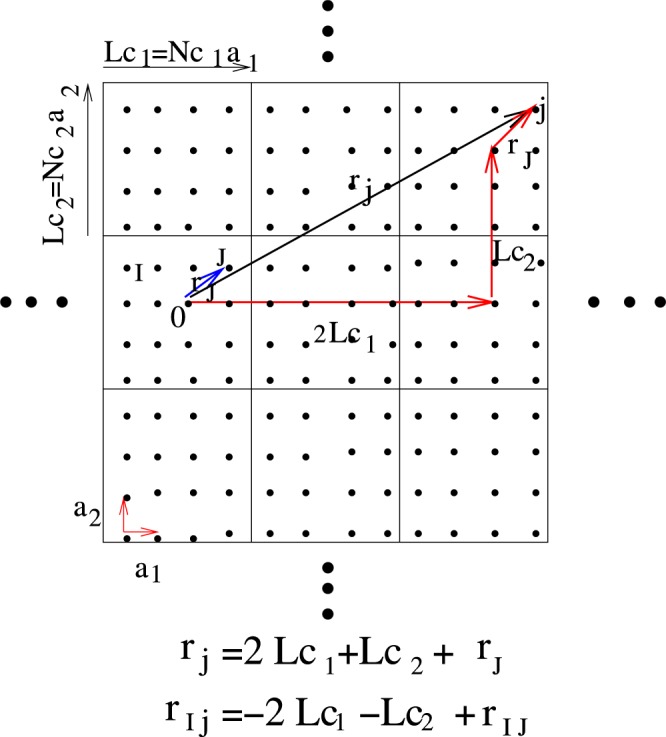
Show a two dimensional square lattice which is divided to similar supercells of *N*_*c*_ = 16 with original lattice symmetry. The super cell vectors are **Lc**_1_ = 4*a***e**_*x*_ and **Lc**_2_ = 4*a***e**_*y*_ where {**a**_1_ = *a***e**_*x*_, **a**_2_ = *a***e**_*y*_} and *a* is lattice constant.

The electron equation of motion for Hamiltonian, Eq. 1, is given by,

2 $$\sum _{l}\,((E-{\varepsilon }_{i}+\mu ){\delta }_{il}-{t}_{il})G(l,j;E)={\delta }_{ij}$$

where *G*(*i*, *j*) is the random single particle Green function. Relation between electron’s random Green function and average Green function is given in Appendix A. Note that although Eqs A.1–A.7 are exact, due to randomness no exact solutions exists. For calculation of self-energy, different single site approximations such as coherent potential approximation (CPA), T-matrix, and Born approximation are introduced. Although attempts have been made with DCA to include multi-site scattering, its coarse grained self energies are discontinuous therefore in k-space these attempts have been unsuccessful. A real space multi site approximation which preserves continuity of k-space dependence of self-energy in the FBZ is not introduced. Here we implement a real space cluster approximation beyond super cell approximation in which not only includes multi site scattering but in the FBZ k-space dependence of self-energy varying continuously. Consider a lattice with lengths {**L**_1_ = *N*_1_**a**_1_, **L**_2_ = *N*_2_**a**_2_, **L**_3_ = *N*_3_**a**_3_ and sites number *N* = *N*_1_*N*_2_*N*_3_ where **a**_*j*_ are lattice primitive vectors. Divide this lattice to super cells with length {**Lc**_1_ = *N*_*c*1_**a**_1_, **Lc**_2_ = *N*_*c*2_**a**_2_, **Lc**_3_ = *N*_*c*3_**a**_3_}, original lattice symmetries and super cell lattice sites number *N*_*c*_ = *N*_*c*1_*N*_*c*2_*N*_*c*3_. Position of *N*_*c*_ sites in side of each cell denoted by capital letters {*I*}. Number of super cells is $\frac{N}{{N}_{c}}$. Since for alloy system at the band splitting regime for *c* = 0.5 and average band filling $\bar{n}=1$ all sites with onsite energy *δ* are empty while sites with onsite energy −*δ* are filled by two electrons, just super cell with even sites number are acceptable. Figure 1 shows this for a two dimensional square lattice with *N*_*c*_ = 16.

Since real space self energies only depend on difference of two lattice sites positions Σ(*E*; *i*, *j*) = Σ(*i* − *j*; *E*), self energies divided to two categories, first self energies between intra sites of each super cell, Σ(*I* − *J*; *E*), second self energies of one site inside of a super cell but another site belongs to another super cell Σ(*I* − *j*; *E*) in which

3 $${{\bf{r}}}_{j}=l{\bf{L}}{{\bf{c}}}_{1}+m{\bf{L}}{{\bf{c}}}_{2}+n{\bf{L}}{{\bf{c}}}_{3}+{{\bf{r}}}_{J}.$$

where $l=\{0,\,1,\mathrm{...},\frac{{N}_{1}}{{N}_{c1}}-1\}$, $m=\{0,1,\mathrm{...},\frac{{N}_{2}}{{N}_{c2}}-1\}$, $n=\{0,1,\mathrm{...},\frac{{N}_{3}}{{N}_{c3}}-1\}$ are integer numbers. The exact **q** space self-energy Σ(**q**; *E*) is

4 $${\rm{\Sigma }}({\bf{q}};E)=\frac{1}{{N}_{c}}\sum _{IJ}\,{\rm{\Sigma }}\,(i,j;E){e}^{i{\bf{q}}.{{\bf{r}}}_{ij}}+\sum _{{q}^{{\rm{^{\prime} }}}}\,{\rm{\Sigma }}\,({{\bf{q}}}^{{\rm{^{\prime} }}};E)\frac{1}{{N}_{c}N}\sum _{IJ}\,{e}^{i({\bf{q}}-{{\bf{q}}}^{{\rm{^{\prime} }}}).{{\bf{r}}}_{IJ}}{{\rm{\Pi }}}_{j=1}^{3}\,(\frac{1-{e}^{-i{N}_{j}{a}_{j}({q}_{j}-{{q}^{{\rm{^{\prime} }}}}_{j})}}{1-{e}^{-i{N}_{cj}{a}_{j}({q}_{j}-{{q}^{{\rm{^{\prime} }}}}_{j})}}-1)$$

details of derivation given in appendix B. Our first approximation for self-energy is that in k-space, contribution of summation over all two lattice sites in different super cells become zero,

5 $$\sum _{{\bf{q}}^{\prime} }\,{\rm{\Sigma }}\,({\bf{q}}^{\prime} ;E)\sum _{IJ}\,{e}^{i({\bf{q}}-{\bf{q}}^{\prime} \mathrm{).}{{\bf{r}}}_{IJ}}{{\rm{\Pi }}}_{j=1}^{3}(\frac{1-{e}^{-i{N}_{j}{a}_{j}({q}_{j}-{q^{\prime} }_{j})}}{1-{e}^{-i{N}_{cj}{a}_{j}({q}_{j}-{q^{\prime} }_{j})}}-1)=0$$

The Born von Karman periodic boundary condition^11^ imply that ${{e}}^{-i{N}_{j}{a}_{j}({q}_{j}-{q^{\prime} }_{j})}=1$, hence in Eq. 5 we have

6 $${e}^{-i{N}_{cj}{a}_{j}({q}_{j}-{q^{\prime} }_{j})}=1.$$

From Eq. 6 we have

7 $${N}_{cj}{{\bf{a}}}_{j}.{{\bf{q}}}_{j}=2\pi {n}_{j},\,j=1,2,3.$$

where {*n*_*j*_} are integer numbers such that one of **q** must be center of FBZ. Wave vectors **q** = **K**_*n*_ that satisfy Eq. 7 are

8 $${{\bf{K}}}_{n}={{\bf{K}}}_{{m}_{1}{m}_{2}{m}_{3}}=\sum _{i=1}^{3}\,\frac{{m}_{i}}{{N}_{ci}}{{\bf{b}}}_{i}.$$

where {**b**_1_, **b**_2_, **b**_3_} are reciprocal lattice primitive vectors, {*m*_1_, *m*_2_, *m*_3_} are integer such that **K**_*n*_ remains in the FBZ. Note that {Σ(**K**_1_; *E*), ..., Σ(**K**_*Nc*_; *E*)} are discontinues in the first Brillouin zone. For *N*_*c*_ = 1, **K**_*n*_ = 0 hence converts to CPA self-energy which is k-independent. It is exact k-space self energy for ${{\rm{l}}{\rm{i}}{\rm{m}}}_{{N}_{c}\to N;I,J\to i,j}\,\frac{1}{{N}_{c}}{\sum }_{IJ}\,{e}^{i{{\bf{K}}}_{n}.{{\bf{r}}}_{IJ}}{\rm{\Sigma }}\,(I,J;E)\,=$ $\frac{1}{N}{\sum }_{ij}{e}^{i{\bf{k}}.{{\bf{r}}}_{ij}}{\rm{\Sigma }}(i,j;E)={\rm{\Sigma }}({\bf{k}};E)$.

**Figure 2:**
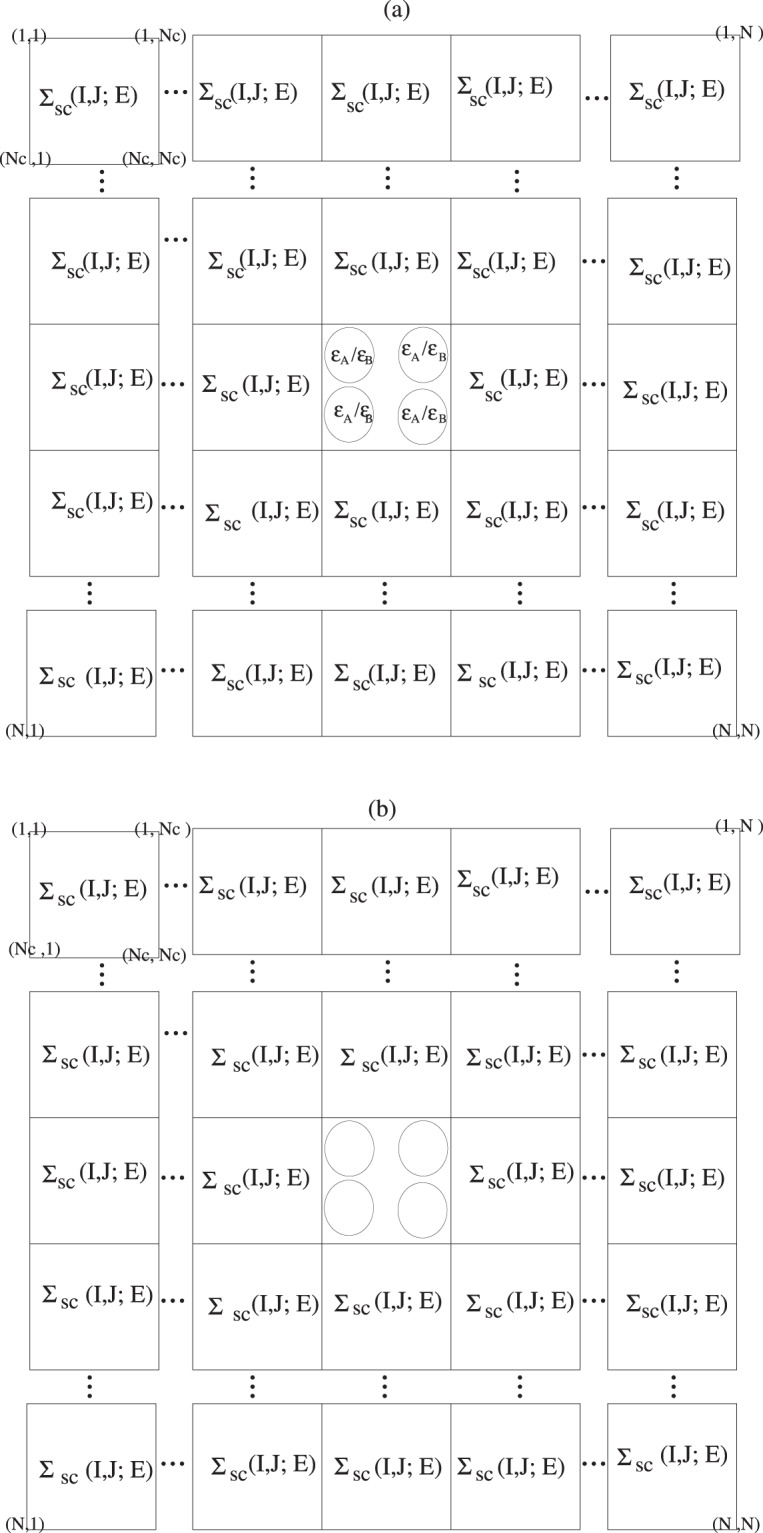
(**a**) Shows an *N*_*c*_ = 4 impurity supercell in an effective supercell self-energy medium in the lattice sites space matrix. (**b**) a *N*_*c*_ = 4 cavity super cell embedded in supercell self energies in real space lattice sites matrix.

By using Eqs 3 and 8 and **a**_*j*_ . **b**_*i*_ = 2*πδ*_*ij*_ hence **K**_*n*_ . *N*_*cj*_**a**_*j*_ = 2*πm*_*j*_ real space self energies of two sites *I* and *j* in different super cells are given by

9 $$\begin{array}{ccc}{\rm{\Sigma }}(I,j;E) & = & \frac{1}{{N}_{c}}\sum _{{K}_{n}}\,{\rm{\Sigma }}\,({{\bf{K}}}_{n};E){e}^{-i{{\bf{K}}}_{n}.{{\bf{r}}}_{IJ}}{e}^{-il{N}_{c1}{{\bf{a}}}_{1}.{{\bf{K}}}_{n}}{e}^{-im{N}_{c2}{{\bf{a}}}_{2}.{{\bf{K}}}_{n}}{e}^{-in{N}_{c3}{{\bf{a}}}_{3}.{{\bf{K}}}_{n}}\\  & = & \frac{1}{{N}_{c}}\sum _{{K}_{n}}\,{\rm{\Sigma }}\,({{\bf{K}}}_{n};E){e}^{-i{{\bf{K}}}_{n}.{{\bf{r}}}_{IJ}}\\  & = & {{\rm{\Sigma }}}_{sc}(I,J;E)\end{array}$$

which are periodic with respect to super cells center vector position. Hence in lattice sites, space self-energy matrix is constructed from just super cell self-energy matrices. This illustrated in Fig. 2.

To calculate Σ(**K**_*n*_; *E*) the FBZ is divided into *N*_*c*_ regions with FBZ symmetries and $\frac{N}{{N}_{c}}$ wave vectors where each of **K**_*n*_ are in the center of one of these grains.Inside each grain self-energy is k-independent therefore, it is grain CPA self-energy. At ${\mathrm{lim}}_{{N}_{c}\to N}$ number of wave vectors {**k**} in each grain reduces to just one (**K**_*n*_ = **k**). The *n* th grain CPA average Green function is defined by

10 $$\bar{G}({{\bf{K}}}_{n};E)=\frac{{N}_{c}}{N}\sum _{{\bf{k}}\in nth\,grain}\,\frac{1}{E-{\varepsilon }_{k}+\mu -\Sigma ({{\bf{K}}}_{n};E)}\mathrm{.}$$

and its real space Fourier transform is

11 $$\bar{G}(I,J;E)=\frac{1}{{N}_{c}}\sum _{{{\bf{K}}}_{n}}\,\bar{G}({{\bf{K}}}_{n};E){e}^{-i{{\bf{K}}}_{n}\mathrm{.}{{\bf{r}}}_{IJ}}\mathrm{.}$$

By taking impurity average over all random lattice sites except central super cell sites, Eq. A.7 reduces to a *N*_*c*_ × *N*_*c*_ matrix of super cell impurity embedded is an effective medium of super cell self energies

12 $${{\bf{G}}}_{{N}_{c}\times {N}_{c}}^{im}={\bar{{\bf{G}}}}_{{N}_{c}\times {N}_{c}}+{\bar{{\bf{G}}}}_{{N}_{c}\times {N}_{c}}{(\varepsilon -{{\rm{\Sigma }}}_{sc})}_{{N}_{c}\times {N}_{c}}{{\bf{G}}}_{{N}_{c}\times {N}_{c}}^{im}.$$

as illustrated in Fig. 2(a). Eq. 12 can be written as

13 $${\bar{{\bf{G}}}}_{sc}^{-1}-{{\rm{\Sigma }}}_{sc}={{\bf{G}}}_{sc}^{im-1}-\varepsilon ={\mathscr{G}}\mathrm{.}$$

where ${\mathscr{G}}$ is called cavity super cell Green function as shown in 2(b). Eq. 13 separates to two following super cell Dysons like equations

14 $${G}_{sc}^{im}(I,J;E)={\mathscr{G}}(I,J;E)+\sum _{L}\,{\mathscr{G}}(I,L;E){\varepsilon }_{L}{G}_{sc}^{im}(L,J;E).$$

and

15 $${\bar{G}}_{sc}(I,J;E)={\mathscr{G}}(I,J;E)+\sum _{LL^{\prime} }\,{\mathscr{G}}(I,L;E){{\rm{\Sigma }}}_{sc}\,(L,L^{\prime} ;E)\bar{G}(L^{\prime} ,J;E).$$

The Fourier transform of real space super cell average Green function, cavity Green function and self-energy to super cell wave vectors {**K**_*n*_} and vice versa are

16 $$\bar{G}({{\bf{K}}}_{n};E)=\frac{1}{{N}_{c}}\sum _{IJ}\,{\bar{G}}_{sc}(I,J;E){e}^{-i{{\bf{K}}}_{n}.{{\bf{r}}}_{IJ}},\,{\bar{G}}_{sc}(I,J;E)=\frac{1}{{N}_{c}}\sum _{{K}_{n}}\,\bar{G}({{\bf{K}}}_{n};E){e}^{i{{\bf{K}}}_{n}.{{\bf{r}}}_{IJ}}$$

17 $${\mathscr{G}}({{\bf{K}}}_{n};E)=\frac{1}{{N}_{c}}\sum _{IJ}\,{\mathscr{G}}(I,J;E){e}^{-i{{\bf{K}}}_{n}\mathrm{.}{{\bf{r}}}_{IJ}},\,{\mathscr{G}}(I,J;E)=\frac{1}{{N}_{c}}\sum _{{K}_{n}}\,{\mathscr{G}}({{\bf{K}}}_{n};E){e}^{i{{\bf{K}}}_{n}\mathrm{.}{{\bf{r}}}_{IJ}}$$

and

18 $${\rm{\Sigma }}({{\bf{K}}}_{n};E)=\frac{1}{{N}_{c}}\sum _{IJ}\,{{\rm{\Sigma }}}_{sc}(I,J;E){e}^{-i{{\bf{K}}}_{n}\mathrm{.}{{\bf{r}}}_{IJ}},\,{{\rm{\Sigma }}}_{sc}(I,J;E)=\frac{1}{{N}_{c}}\sum _{{K}_{n}}\,{\rm{\Sigma }}\,({{\bf{K}}}_{n};E){e}^{i{{\bf{K}}}_{n}\mathrm{.}{{\bf{r}}}_{IJ}}$$

respectively. Using following relation

19 $$\frac{1}{{N}_{c}}\sum _{I}{e}^{i({{\bf{K}}}_{n}-{{\bf{K}}}_{n^{\prime} }).{{\bf{r}}}_{I}}={\delta }_{{{\bf{K}}}_{n}{{\bf{K}}}_{n^{\prime} }},\,\frac{1}{{N}_{c}}\sum _{{{\bf{K}}}_{n}}\,{e}^{-i{{\bf{K}}}_{n}.{{\bf{r}}}_{IJ}}={\delta }_{IJ}$$

Substituting Eqs 16–18 in Eq. 15 and using Eq. 19 we have

20 $$\bar{G}({{\bf{K}}}_{n};E)={\mathscr{G}}({{\bf{K}}}_{n};E)+{\mathscr{G}}({{\bf{K}}}_{n};E){\rm{\Sigma }}({{\bf{K}}}_{n};E)\bar{G}({{\bf{K}}}_{n};E).$$

## Beyond Supercell Approximation

To go beyond supercell approximation and add summation of self energies contribution of all sites *i* and *j* which in real space are not in the same supercell we use supercell approximation for ${\rm{\Sigma }}(I,J;E),{\rm{\Sigma }}(I,J;E)\approx {{\rm{\Sigma }}}_{sc}(I,J;E)\,=$ $\frac{1}{{N}_{c}}{\sum }_{{{\bf{K}}}_{n}}\,{\rm{\Sigma }}\,({{\bf{K}}}_{n};E){e}^{i{{\bf{K}}}_{n}\mathrm{.}{{\bf{r}}}_{IJ}}$ hence

21 $$\sum _{IJ}\,{\rm{\Sigma }}\,(I,J;E){e}^{i{\bf{k}}\mathrm{.}{{\bf{r}}}_{IJ}}\approx \sum _{IJ}\,{{\rm{\Sigma }}}_{sc}\,(I,J;E){e}^{i{\bf{k}}\mathrm{.}{{\bf{r}}}_{IJ}}=\frac{1}{{N}_{c}}\sum _{IJ}\,\sum _{{{\bf{K}}}_{n}}\,{\rm{\Sigma }}\,({{\bf{K}}}_{n};E){e}^{i({\bf{k}}-{{\bf{K}}}_{n}\mathrm{).}{{\bf{r}}}_{IJ}}\mathrm{.}$$

Note that beyond supercell approximation where 1 < *N*_*c*_ < *N*, for **q**_*j*_ ≠ **K**_*nj*_ we have $1-{e}^{-i{N}_{cj}{a}_{j}({q}_{j}-{q^{\prime} }_{j})}\ne 0$. By inserting Eq. 21 in to Eq. B.4 we have

22 $${\rm{\Sigma }}({\bf{k}};E)=\frac{1}{{N}_{c}^{2}}\sum _{IJ}\,\sum _{{{\bf{K}}}_{n}}\,{\rm{\Sigma }}\,({{\bf{K}}}_{n},E){e}^{i({\bf{k}}-{{\bf{K}}}_{n}).{{\bf{r}}}_{IJ}}-\sum _{{\bf{q}}^{\prime} }\,{\rm{\Sigma }}\,({\bf{q}}^{\prime} ;E)\frac{1}{{N}_{c}N}\sum _{IJ}{e}^{i({\bf{k}}-{\bf{q}}^{\prime} ).{{\bf{r}}}_{IJ}}$$

Eq. 22 is centerpiece of our approximation. By iteration, Eq. 22 up to first order reduces to

23 $${\rm{\Sigma }}({\bf{k}};E)=\frac{1}{{N}_{c}^{2}}\sum _{IJ}\,\sum _{{{\bf{K}}}_{n}}\,{\rm{\Sigma }}\,({{\bf{K}}}_{n};E){e}^{i({\bf{k}}-{{\bf{K}}}_{n}).{{\bf{r}}}_{IJ}}(1-\frac{1}{{N}_{c}})$$

where 1 < *N*_*c*_ < *N*. Table 1 shows comparison of **k**-space self energies of various approximations, single site coherent potential approximation (CPA) with k-independent self energy Σ(**k**; *E*) = Σ(*E*), dynamical cluster approximation (DCA) and supercell approximations Σ(**k**; *E*) = {Σ(**K**_1_; *E*), ..., Σ(**K**_*Nc*_; *E*)} which are cluster wave vectors {**K**_1_, ..., **k**_*Nc*_} dependent and beyond supercell approximation ${\rm{\Sigma }}({\bf{k}};E)=\frac{1}{{N}_{c}^{2}}{\sum }_{IJ}\,{\sum }_{{{\bf{K}}}_{n}}\,{\rm{\Sigma }}\,({{\bf{K}}}_{n};E){e}^{i({\bf{k}}-{{\bf{K}}}_{n}\mathrm{).}{{\bf{r}}}_{IJ}}(1-\frac{1}{{N}_{c}})$ which is fully k-dependent in the first Brillouin zone.

**Table 1:** Shows comparison of k-space self energy in various approximations.

Approximation	Self energy Σ(k; *E*) =	Dependence on k in FBZ
CPA	Σ(*E*)	**k** independent
DCA and supercell approximation	{Σ(**K**_1_; *E*), ..., Σ(**K**_*Nc*_; *E*)}	**k** = {**K**1, ..., **k**_*Nc*_}
beyond supercell approximation	$\frac{1}{{N}_{c}^{2}}{\sum }_{IJ}\,{\sum }_{{K}_{n}}\,{\rm{\Sigma }}({{\bf{K}}}_{n};E){e}^{i({\bf{k}}-{{\bf{K}}}_{n}).{{\bf{r}}}_{IJ}}(1-\frac{1}{{N}_{c}})$	all **k** in the first Brillouin zone

For calculation of self energy Σ(**k**; *E*) in Eq. 23 first we calculate Σ(**K**_*n*_; *E*). Algorithm for calculation of average Green function, $\bar{G}({\bf{k}};E)$, is as follows

1. A guess is made for real space and k-space self energies, Σ(*I*, *J*; *E*), and Σ(**K**_*n*_). The starting values are usually zero.
2. By inserting Σ_*sc*_(**K**_*n*_; *E*) in Eq. 10, $\bar{G}({{\bf{K}}}_{n};E)=\frac{{N}_{c}}{N}{\sum }_{{\bf{k}}\in nthgrain}{({G}_{0}^{-1}({\bf{k}};E)-{\rm{\Sigma }}({{\bf{K}}}_{n};E))}^{-1}$, calculate the grain average k-space Green functions, $\bar{G}({{\bf{K}}}_{n};E)$.
3. From Eq. 20 calculate K-space cavity Green function ${\mathscr{G}}({{\bf{K}}}_{n};E)={({\bar{G}}^{-1}({{\bf{K}}}_{n};E)+{\rm{\Sigma }}({{\bf{K}}}_{n};E))}^{-1}$.
4. Obtain real space cavity Green function ${\mathscr{G}}(I,J;E)=\frac{1}{{N}_{c}}{\sum }_{{{\bf{K}}}_{n}}\,{e}^{i{{\bf{K}}}_{n}\mathrm{.}{{\bf{r}}}_{IJ}}{\mathscr{G}}({{\bf{K}}}_{n};E)$ by Fourier transform of k-space ${\mathscr{G}}({{\bf{K}}}_{n};E)$.
5. Calculate real space super cell impurity Green function matrix ${G}^{imp}={({{\mathscr{G}}}^{-1}-\varepsilon )}^{-1}$.
6. Calculate supercell impurity average Green function matrix $\bar{G}(I,J;E)= < ({{\mathscr{G}}}^{-1}-{\boldsymbol{\varepsilon }}{)}^{-1}{ > }_{IJ}$ by taking average over all possible impurity configurations.
7. Calculate real space new supercell self-energy matrix from ${{\rm{\Sigma }}}_{sc}={{\mathscr{G}}}^{-1}-{\bar{G}}^{-1}$.
8. Inverse Fourier transform of new average supercell self-energy to calculate $$\sum ({{\bf{K}}}_{n};E)\,=$$$$\frac{1}{{N}_{c}}{\sum }_{IJ}\,{e}^{-i{{\bf{K}}}_{n}\mathrm{.}{{\bf{r}}}_{IJ}}{{\rm{\Sigma }}}_{sc}\,(I,J;E)$$.
9. Return to 2 and repeat until convergence.
10. Calculate self-energy beyond supercell approximation, Σ(**k**; *E*), by substitution obtained Σ(**K**_*n*_; *E*) in Eq. 23.
11. Calculate k-space average green function from $\bar{G}({\bf{k}};E)={G}_{0}^{-1}({\bf{k}};E)-{\rm{\Sigma }}({\bf{k}};E)$.
12. Calculate real space average green function from $\bar{G}(i,j;E)=\frac{1}{N}{\sum }_{{\bf{k}}}\,({G}_{0}^{-1}({\bf{k}};E)-{\rm{\Sigma }}({\bf{k}};E)){e}^{i{\bf{k}}.{{\bf{r}}}_{ij}}$.
13. Calculate beyond super cell real space self energy from ${\rm{\Sigma }}(i,j;E)=\frac{1}{N}{\sum }_{{\bf{k}}}\,{\rm{\Sigma }}\,({\bf{k}};E){e}^{i{\bf{k}}.{{\bf{r}}}_{ij}}$.

**Figure 3:**
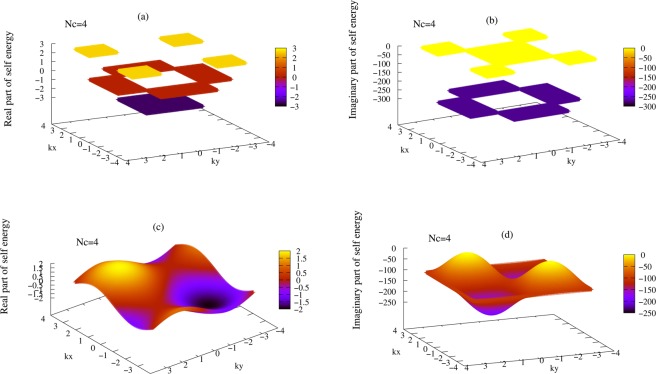
(**a**,**b**) Show real and imaginary parts of supercell self energies $\frac{1}{4t}{\rm{\Sigma }}\,({{\bf{K}}}_{n},\,0+i\eta )$ for the *N*_*c*_ = 4 of a two dimensional square alloy. (**c**,**d**) Shows real and imaginary parts of self energy $\frac{1}{4t}{\rm{\Sigma }}({\bf{k}},\,0+i\eta )$ of a two dimensional square alloy system in beyond *N*_*c*_ = 4 supercell approximation for *δ* = 3*t*, *c* = 0.5 and *μ* = 0. In the supercell approximation k-space self energy in FBZ is discontinuous and k-independent in each grain, but in our beyond supercell approximation it is continuous and fully k-dependent.

**Figure 4:**
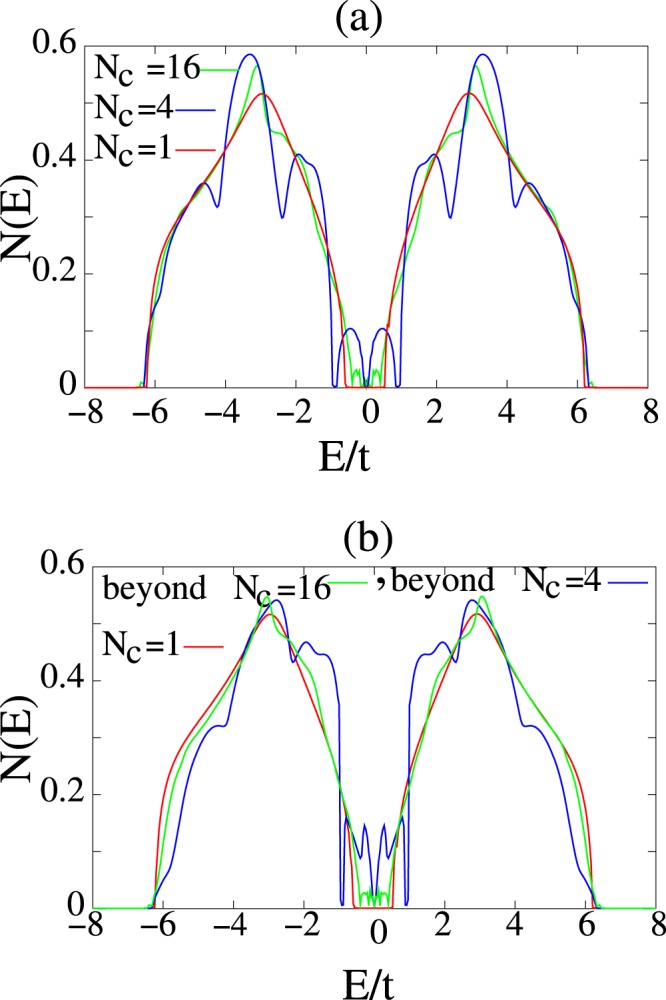
Show comparison of average density of states of a two dimensional square alloy system for (**a**) CPA *N*_*c*_ = 1, supercell approximation *N*_*c*_ = 4, *N*_*c*_ = 16 and (**b**) beyond *N*_*c*_ = 4 and *N*_*c*_ = 16 supercell approximation. The strength length, *δ* = 3*t*, impurity concentration is *c* = 0.5 and *μ* = 0. The difference between density of states are due to nonlocal corrections.

Although this method is general but we apply this method to a two dimensional square alloy system in which *δ* = 3*t*, *c* = 0.5 and *μ* = 0. For this system we calculate, self-energy and density of states in the supercell and beyond super cell approximations and compared them. Figure 3(a,b) shows real and imaginary part of self energy Σ(**K**_*n*_; 0) in terms of *k*_*x*_ and *k*_*y*_ in supercell approximation for *N*_*c*_ = 4. self-energy at the borders of grains have discontinuity and inside of each grain is k-independent. (c) and (d) shows real and imaginary parts of self-energy Σ(**k**; 0) in the beyond *N*_*c*_ = 4 supercell approximation which are fully k-dependent and causal. Figure 4(a) shows calculated average density of states for supercells *N*_*c*_ = 1, *N*_*c*_ = 4, and *N*_*c*_ = 16 for *δ* = 3*t*, *c* = 0.5 and band filling $\bar{n}=1$. (b) shows average density of states calculated by our beyond supercell approximation for *N*_*c*_ = 1, *N*_*c*_ = 4, and *N*_*c*_ = 16. However bands of this system in this regime splitted in CPA and super cell approximation but in our approximation beyond supercell it is at beginning of splitting^12^.

One of advantage of supercell approximation is to take into account electron localization in one and two dimensional disordered alloys which calculates by^13,14^

24 $$P({\rm{\infty }})=li{m}_{t\to {\rm{\infty }}} < |{G}_{ll}^{im}(t){|}^{2} > =li{m}_{\eta \to 0}\int dE < |{G}_{ll}^{im}(E+i\eta ){|}^{2} > $$

The random local green function *G*_*ll*_(*ε* + *iη*) in the CPA is ${G}_{cpa\,ll}^{im}(E+i\eta )=({{\mathscr{G}}}_{ll}^{-1}(E+i\eta )-\varepsilon +{{\rm{\Sigma }}}_{cpa}{)}^{-1}$, but in the super cell approximation is ${G}_{sc\,ll}^{im}(E+i\eta )={({{\mathscr{G}}}^{-1}-\varepsilon +{{\rm{\Sigma }}}_{sc})}_{ll}^{-1}$ and finally in the beyond super cell approximation is ${G}_{bsc\,ll}^{im}(E+i\eta )={({\bar{{\bf{G}}}}^{-1}-{\boldsymbol{\varepsilon }}+{\boldsymbol{\Sigma }})}_{ll}^{-1}$ where ${{\boldsymbol{\Sigma }}}_{l{l}^{{\rm{^{\prime} }}}}(E+i\eta )=\frac{1}{N}{\sum }_{{\bf{k}}}\,{\rm{\Sigma }}\,({\bf{k}};E){e}^{ik.{r}_{l{l}^{^{\prime} }}}$. After calculation of local impurity green function and substitution it in Eq. 24 localization *P*(∞) obtains.

**Figure 5:**
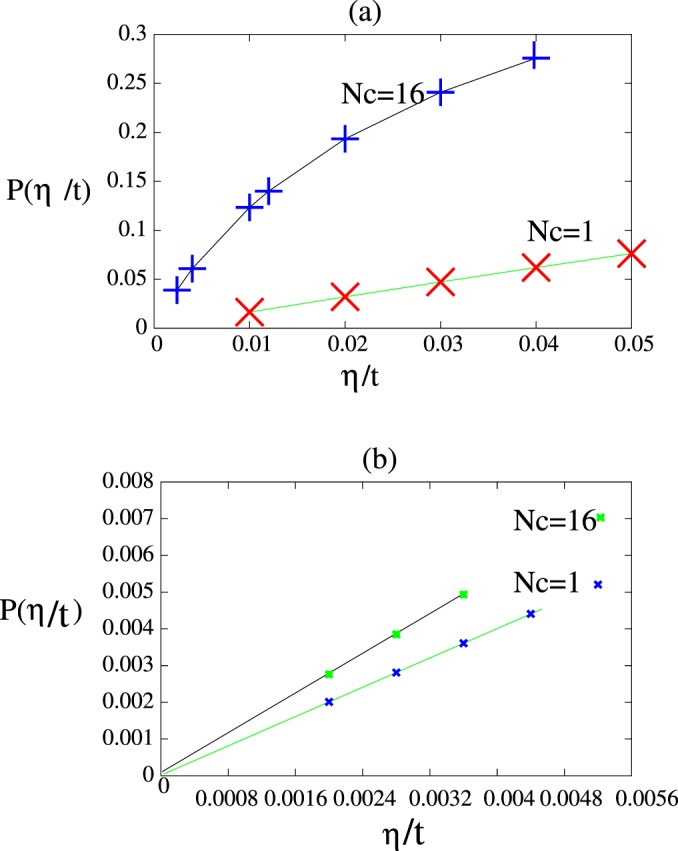
Shows electron localization probability at site *l* for: (**a**) one dimensional alloy for *δ* = 3*t*, *c* = 0.5 and average band filling $\bar{n}=1$. For *N*_*c*_ = 1, $P(\frac{\eta }{t})$ extrapolated to zero while for *N*_*c*_ = 16 is fitted to $P(\frac{\eta }{t})=0.007613+14.21(\frac{\eta }{t})-300.7{(\frac{\eta }{t})}^{2}+28.28{(\frac{\eta }{t})}^{3}$. Hence probability of localization is *P*(0) = 0.007613. (**b**) a two dimensional square alloy system for CPA *N*_*c*_ = 1 and super cell approximation *N*_*c*_ = 16. The strength length, *δ* = 3*t*, impurity concentration is *c* = 0.5 and *μ* = 0. CPA doesn’t shows localization but for *N*_*c*_ = 16 super cell, $P(\frac{\eta }{t}=0)=2.45\times {10}^{-5}$ which is due to electron back scattering in the super cell.

Figure 5 shows probability of remaining electron at site *l* for (a) a one dimensional lattice in the CPA and *N*_*c*_ = 16 super cell approximations for *δ* = 3*t*, *c* = 0.5 and *μ* = 0.

CPA $$P(\frac{\eta }{t})$$ extrapolated to zero while for *N*_*c*_ = 16 it is fitted by $$P(\frac{\eta }{t})=0.007613+14.21(\frac{\eta }{t})-300.7{(\frac{\eta }{t})}^{2}+$$$$28.28{(\frac{\eta }{t})}^{3}$$ hence *P*(0) = 0.007613. (b) shows it for a square two dimensional alloy in the CPA and *N*_*c*_ = 16 super cell approximation. In the CPA it is extrapolates to zero but for *N*_*c*_ = 16 it is extrapolating to non zero value $$P(\frac{\eta }{t}=0)=2.45\times {10}^{-4}$$.

## Conclusion

For investigating metal-insulator phase transition due to electron localization in disordered alloys and interaction electrons systems a successful multi site approximation beyond supercell approximation introduced. In this approximation self-energy is casual and full k-dependent in the first Brillouin zone. For derivation of the approximation, the entire lattice is divided to supercells with *N*_*c*_ sites and no overlap. We proved that self-energy with one site in a definite super cell but another in other supercells are periodic with respect to supercell lengths. Correction to k-space supercell self-energy comes from sites in different super cells. We added this part to the k-space supercell self-energy. Our approximation recovers CPA in the single site cell limit and as the number of supercell sites approaches the number of lattice sites, *N*_*c*_ → *N*, becomes exact. This approximation opens a new channel for observing multi sites scattering effects such as localization that are not observed by other previous approximations. It is overcomes discontinuity and weakly k-dependent of previous approximations where especially for low dimensional systems self energy is k-dependent significantly. By applying this method to one and two dimension alloy electron localization observed.

## Supplementary information

supplementary
