# Supplementary material for: Low dimensions electron localization in the beyond real space super cell approximation
Source: Sci Rep. 2019 Jun 4;9:8288. doi: 10.1038/s41598-019-44395-w (PMC6547744; doi:10.1038/s41598-019-44395-w)
Supplement: Supplementary file 1 — supplementary [file 41598_2019_44395_MOESM1_ESM.pdf]

Low dimensions electron localization in the beyond real space super cell approximation.

Rostam Moradian[1,2,*] **and Sina Moradian**[3]

[1]Department of Physics, Faculty of Science Razi University, Kermanshah, Iran
[2]Nano science and nano technology research center, Razi University, Kermanshah, Iran
[*]Correspondence and requested materials should be addressed to R. M. [rmoradian@razi.ac.ir]
[3]Department of Electrical and Computer Engineering, University of Central Florida, Orlando, Florida, USA.

Supplementary Materials:

APPENDIX A: Relation between random and average effective medium Green functions.
APPENDIX B: K-space self-energy contribution of two sites inside super cells or one intra while other is on the other super cells.

A Relation between random and average effective medium Green functions

By writing Eq.2 in matrix form in lattice sites space it could be rewritten as

$$G(i,j;E) = G^0(i,j;E) + \sum_l G^0(i,l;E)\varepsilon_l G(l,j;E). \tag{A.1}$$

The Dysons equation corresponding to Eq.2 for the exact averaged Green function, $\bar{G}(l,j)$, is[1],

$$\bar{G}(i,j;E) = G^0(i,j;E) + \sum_{ll'} G^0(i,l;E)\Sigma(l,l';E)G(l',j;E) \tag{A.2}$$

where the real lattice sites space self-energy matrix, Σ, is,

$$\langle \varepsilon \mathbf{G} \rangle = \Sigma \bar{\mathbf{G}}. \tag{A.3}$$

Eq.A.2 in k-space is

$$\bar{G}(\mathbf{k};E) = (G_0^{-1}(E;\mathbf{k}) + \Sigma(\mathbf{k};E))^{-1} \tag{A.4}$$

where

$$\bar{G}(\mathbf{k};E) = \frac{1}{N}\sum_{ij} e^{i\mathbf{k}.\mathbf{r}_{ij}} \bar{G}(i,j;E) \tag{A.5}$$

and

$$\Sigma(\mathbf{k};E) = \frac{1}{N}\sum_{ij} \Sigma(i,j;E) e^{i\mathbf{k}.\mathbf{r}_{ij}} \tag{A.6}$$

Combining Eqs. A.1 and A.2 relation between lattice sites space random Green function and lattice sites average Green function matrix is

$$\mathbf{G} = \bar{\mathbf{G}} + \bar{\mathbf{G}}(\varepsilon - \Sigma)\mathbf{G} \tag{A.7}$$

B K-space self-energy contribution of two sites inside super cells or one intra while other is on the other super cells.

In k-space

$$\Sigma(\mathbf{q};E) = \frac{1}{N}\sum_{ij}\Sigma(i,j;E)e^{i\mathbf{q}.\mathbf{r}_{ij}} = \frac{1}{N}\sum_{ij\in\text{same super cell}}\Sigma(i,j;E)e^{i\mathbf{q}.\mathbf{r}_{ij}} + \frac{1}{N}\sum_{ij\notin\text{same super cell}}\Sigma(i,j;E)e^{i\mathbf{q}.\mathbf{r}_{ij}} \tag{B.1}$$

The first term of right hand side of Eq. B.1 is number of supercell, $\frac{N}{N_c}$, times one supercell contribution and second term equals to number of supercell times self-energy of one site in a supercell but other site in whole supercells

$$\Sigma(\mathbf{q};E) = \frac{1}{N_c} \sum_{IJ \in \text{same supercell}} \Sigma(I,J;E) e^{i\mathbf{q}.\mathbf{r}_{IJ}} + \frac{1}{N_c} \sum_{Ij \notin \text{same supercell}} \Sigma(I,j;E) e^{i\mathbf{q}.\mathbf{r}_{Ij}} \tag{B.2}$$

We rewrite Eq. B.2 as

$$\Sigma(\mathbf{q};E) = \frac{1}{N_c} \sum_{IJ \in \text{same supercell}} \Sigma(I,J;E) e^{i\mathbf{q}.\mathbf{r}_{IJ}} + \frac{1}{N_c N} \sum_{Ij \notin \text{same supercell}} \sum_{\mathbf{q}'} \Sigma(\mathbf{q}';E) e^{-i\mathbf{q}'.\mathbf{r}_{Ij}} e^{i\mathbf{q}.\mathbf{r}_{Ij}} \tag{B.3}$$

By inserting Eq. 3 in to Eq. B.3 in terms of $\{I,J\}$ in the same super cell we have

$$\begin{aligned} \Sigma(\mathbf{q};E) &= \frac{1}{N_c} \sum_{IJ} \Sigma(I,J;E) e^{i\mathbf{q}.\mathbf{r}_{IJ}} + \sum_{\mathbf{q}'} \Sigma(\mathbf{q}';E) \frac{1}{N_c N} \sum_{IJ} e^{i(\mathbf{q}-\mathbf{q}').\mathbf{r}_{IJ}} \sum_{m_1 m_2 m_3} e^{i(\mathbf{q}-\mathbf{q}').(m_1 \mathbf{L}_1 + m_2 \mathbf{L}_2 + m_3 \mathbf{L}_3)} \\ &= \frac{1}{N_c} \sum_{IJ} \Sigma(I,J;E) e^{i\mathbf{q}.\mathbf{r}_{IJ}} + \sum_{\mathbf{q}'} \Sigma(\mathbf{q}';E) \frac{1}{N_c N} \sum_{IJ} e^{i(\mathbf{q}-\mathbf{q}').\mathbf{r}_{IJ}} \Pi_{j=1}^{3} \left(\frac{1 - e^{-iN_j a_j (q_j - q'_j)}}{1 - e^{-iN_{cj} a_j (q_j - q'_j)}} - 1 \right) \end{aligned} \tag{B.4}$$

Note that Eq. B.4 is exact.

References

1. Ziman J. M. *Models of Disorder* (Cambridge University Press, Cambridge, England, 1979).
